# A rank based social norms model of how people judge their levels of drunkenness whilst intoxicated

**DOI:** 10.1186/s12889-016-3469-z

**Published:** 2016-09-13

**Authors:** Simon C. Moore, Alex M. Wood, Laurence Moore, Jonathan Shepherd, Simon Murphy, Gordon D. A. Brown

**Affiliations:** 1 Violence & Society Research Group, School of Dentistry, Cardiff University, Cardiff, Wales CF14 4XY UK; 2 Behavioural Science Centre, Stirling Management School, University of Stirling, Stirling, Scotland FK9 4LA UK; 3 MRC/CSO Social and Public Health Sciences Unit, Institute of Health and Wellbeing, University of Glasgow, 200 Renfield Street, Glasgow, Scotland G2 3QB UK; 4 Centre for the Development and Evaluation of Complex Interventions for Public Health Improvement, Cardiff School of Social Sciences, Cardiff University, 1-3 Museum Place, Cardiff, Wales CF10 3BD UK; 5 Department of Psychology, University of Warwick, Coventry, England CV4 7AL UK

**Keywords:** Alcohol, Risk, Social norms, Decision by sampling, Relative rank hypothesis

## Abstract

**Background:**

A rank based social norms model predicts that drinkers’ judgements about their drinking will be based on the rank of their breath alcohol level amongst that of others in the immediate environment, rather than their actual breath alcohol level, with lower relative rank associated with greater feelings of safety. This study tested this hypothesis and examined how people judge their levels of drunkenness and the health consequences of their drinking whilst they are intoxicated in social drinking environments.

**Methods:**

Breath alcohol testing of 1,862 people (mean age = 26.96 years; 61.86 % male) in drinking environments. A subset (*N* = 400) also answered four questions asking about their perceptions of their drunkenness and the health consequences of their drinking (plus background measures).

**Results:**

Perceptions of drunkenness and the health consequences of drinking were regressed on: (a) breath alcohol level, (b) the rank of the breath alcohol level amongst that of others in the same environment, and (c) covariates. Only rank of breath alcohol level predicted perceptions: How drunk they felt (*b* 3.78, 95 % CI 1.69 5.87), how extreme they regarded their drinking that night (*b* 3.7, 95 % CI 1.3 6.20), how at risk their long-term health was due to their current level of drinking (*b* 4.1, 95 % CI 0.2 8.0) and how likely they felt they would experience liver cirrhosis (*b* 4.8. 95 % CI 0.7 8.8). People were more influenced by more sober others than by more drunk others.

**Conclusion:**

Whilst intoxicated and in drinking environments, people base judgements regarding their drinking on how their level of intoxication ranks relative to that of others of the same gender around them, not on their actual levels of intoxication. Thus, when in the company of others who are intoxicated, drinkers were found to be more likely to underestimate their own level of drinking, drunkenness and associated risks. The implications of these results, for example that increasing the numbers of sober people in night time environments could improve subjective assessments of drunkenness, are discussed.

## Background

Excessive alcohol consumption places drinkers’ health at risk both in the long term and during the drinking episode [1]. In the drinking episode intoxication can lead to risk taking behaviours such as unsafe sex, driving a motor vehicle while intoxicated, criminal or social misdemeanors of varying levels of seriousness, and continuation of drinking until ataxia, coma and even death occur [2, 3]. Mis-estimations of the degree of personal intoxication may contribute to such behaviours, leading a person to believe that they are able to undertake a task safely when they are not, or to believe that they can continue drinking without becoming dangerously intoxicated. Understanding how people judge their drunkenness whilst intoxicated, how their current state of intoxication impacts on their health, and how such estimates can be systematically biased, is an important first step towards designing environments and honing interventions to reduce excessive drinking and drunken mis-behaviour. However, whilst much research has focused on how people evaluate the heaviness of their drinking (e.g., [4–7]), this research is normally conducted with participants whilst they are sober, and little is known about how people evaluate their drunkenness whilst actually under the influence of alcohol and in a drinking environment. The importance of this becomes greater where there are concentrations of drinkers, a phenomenon which has increased in UK cities that have been focal points for concentrated development of the night time environment, leading to a high density of licensed premises.

Whilst intoxicated, people might be assumed to judge their levels of drunkenness based solely on how much they have actually drunk. This intuitive “actual intoxication” approach is perhaps a partial motivation for calls to increase the availability of information on the amount of alcohol consumed, for example through mandatory labeling of bottles with alcohol units [8].

We propose in contrast a rank based social norms model, which we test in a sample of intoxicated individuals through modeling the relationship between people’s objective drunkenness (based on breath alcohol concentration, BrAC, measured using an alcometer) and their ratings of their drunkenness, as well as the relationship between their BrAC and the attitudes they held at that moment about the health consequences of their drinking. Specifically we hypothesize that when drinking alcohol amongst others people’s perception of their own level of drunkenness is influenced by the perception of their level of inebriation relative to others in their immediate vicinity. Our focus is on social drinking and we make no reference to alcohol consumption that occurs in isolation. The “actual intoxication” approach neglects existing social norms research which indicates that - at least whilst individuals are sober - people are highly influenced by their perceptions of how their levels of drinking compare to those of others within their reference groups (e.g., [4–7]). Whilst this conclusion has been established for sober individuals, the same may not hold for real world settings (i.e., for intoxicated individuals in drinking environments).

First, it is not clear whether drinkers compare their levels of intoxication to the actual levels of intoxication of those in the same drinking environment, or to their incorrect beliefs about the levels of intoxication of others. Generally, social norms research has shown that people have an inaccurate impression of how much others drink - possibly being motivated by a desire to self-enhance through seeing themselves as relative lower drinkers - and that it is this inaccurate impression that affects judgements of the heaviness of actual drinking [4, 7]. However such research, conducted with sober participants in non-drinking environments requires participants to rely on memory to make comparisons, and this involvement of memory may lead to biased judgements about personal drinking [9]. In contrast, people can actually observe the intoxication levels of others whilst in drinking environments [10], providing opportunities for people to be more influenced by the actual rather than remembered states of others. The physical presence of others may reduce the biasing effect of memory.

Second, it is also not clear whether: (a) comparisons to others would bias the basic relationship between objective and subjective drunkenness (such that subjective drunkenness would be predicted by both objective drunkenness and social comparisons), or (b) the relationship between objective and subjective drunkenness is wholly based on comparisons to others (such that when statistically controlling for social comparisons there would no longer be a relationship between objective and subjective drunkenness).

Third, the cognitive mechanisms through which people compare their level of drinking to that of others are not known. In making specific predictions for this study, we were guided by independent research from psychophysics which focuses on how people judge the magnitude of stimuli (see [11]). Such research is directly relevant as it concerns how people make subjective judgements (here, drunkenness) based on objective magnitudes (here, objective intoxication). Historically, such research has followed a path of initially assuming that people are influenced by the actual magnitude of the stimuli (here, actual intoxication, see [12]), subsequently assuming people are influenced by how a stimulus differs from some measure of central tendency (here, for example, how one’s intoxication differs from the average intoxication within the environment, [13]), and finally showing that people are only sensitive to how a stimuli ranks within the environment (here, how one’s drinking ranks within the immediate environment, see [11]). The rank hypothesis has been supported in a variety of other psychophysiological [14–16] and social [17–21] domains. Such a perspective raises the possibility that individuals in drinking environments may base the estimates of the heaviness of their drinking wholly on how their level of intoxication ranks relative to that of others (rather than rank based comparisons providing an additive bias). Showing that common mechanisms apply in different areas furthers the development of a more unified and integrated psychology [22] where the same cognitive mechanisms are shown to operate across multiple domains.

Fourth, if people do compare themselves to others, it is not clear whether their judgements of their own intoxication would be equally, more, or less influenced by people who drink more than they do relative to those who drink less. The alcohol and social norms literature suggests that sober people have a tendency to over-estimate how much others drink, this effect being consistent with a self-enhancement bias motivated by a desire to see one’s consumption as relatively lower (e.g., [4, 7]). This might suggest that people would be more influenced by those who drink more than they do themselves. However, it is again not clear that findings based on sober individuals in classroom or home settings would generalize straightforwardly to intoxicated individuals in real world environments. In such real world settings more sober people may be more salient, leading to a greater relative comparison to those who have drunk less. It is also not clear which comparisons a self-enhancement bias may predispose; when sober people focus on general alcohol consumption it may seem preferable to drink less, whereas whilst in a “party” mood and intoxicated in a drinking environment it may seem preferable to drink relatively more. Thus the very self-enhancement biases that predispose comparisons to heavier drinking people in sober environments may predispose comparisons to lower level drinkers in real world ones.

In this study we examine for the first time how people judge their drunkenness and the health consequences of their drinking whilst they are intoxicated in social drinking environments. The focus on health was motivated by recent calls for more social norms research to focus on perceptions of the health consequences of personal levels of drinking in addition to simply perceptions of the heaviness of drinking [9, 23]. Based on previous social norms research we hypothesize that such judgements will be influenced by how the individual compares themselves to others. As such others are salient in this environment we further hypothesize that people will be influenced by how the individual’s intoxication ranks amongst the actual levels of intoxication of others in the environment. Finally, based on independent research from cognitive science [11, 12], we hypothesize that judgements will be wholly based on how the individual’s intoxication compares to others in the environment, and that these comparisons will be rank based, arising from the same cognitive mechanisms used to judge psychophysical stimuli. We have developed no specific hypothesis as to whether people will be more influenced by those who are more or less intoxicated and leave this test as exploratory.

## Method

### Participants and procedure

This research was a component of a larger twenty-four month study [24], twelve months of which involved a large scale street survey. The data collection reported here was undertaken in busy night time environments characterised by a high density of premises licensed for the on-site sale and consumption of alcohol. Full project details and instrument validation are published elsewhere [10, 24, 25]. The study was scrutinised and approved by the Cardiff Dental School Research Ethics Committee. The survey involved two pairs of surveyors who approached every seventh individual walking past designated sampling landmarks (central locations through which a high volume of pedestrians would pass). We estimate the median group size was four [26] and therefore selection of every seventh individual would mean we were less likely to consecutively select individuals from the same social group. Individuals were approached and asked to participate. Those who consented breathed into alcometers, calibrated to ±3 μg alcohol/100 ml breath, which recorded respondents’ objective BrAC. Participants were also asked what time they began drinking alcohol that evening (from which session duration was derived), and completed the Fast Alcohol Screening Test (FAST) which is used to assess respondents’ historical levels of risky alcohol consumption [27]. The sampling strategy attempted to ensure that a representative sample was approached and all those approached were deemed eligible to participate unless they were at work (e.g. police officers). Surveys were typically conducted between 8 pm and 3 am on Friday and Saturday evenings. Verbal consent to participate was requested from each participant and all study participants were told that they could stop participating at any time. In total, alcometer reading, gender, and location information were recorded for 1,862 people across four locations (63.2 % were male, consistent with the gender composition of the areas sampled [28]). These participants were part of the wider study programme. In addition, 669 participants were invited to answer additional questions specifically for the current study. Five hundred and thirty four participants (80 %) consented to participate and 477 completed the key four questions (set out below). There were no significant differences in BrAC between those who responded to all four questions and those who completed three or fewer (*t* < 0.1). Of these 477 participants, 39 did not provide gender information and 38 did not answer the covariation questions (e.g., FAST test, time starting drinking etc.). This left a usable study sample of 400 participants who completed all relevant measures (representing a 60 % response rate from those asked to participate).

Four questions were administered to assess perceived drunkenness and potential health risks of the current levels of drinking; (1) *drunkenness* (“how drunk are you right now, on a 1 (totally sober) to 10 (completely drunk) scale?”), (2) *extreme drinking* (“how extreme has your drinking been tonight, on a 1 (not at all) to 10 (completely extreme) scale?”), (3) *risk to long-term health* (“if you drank as much as you have tonight every week how likely is it that you will damage your health in the next 15 years, on a 1 (definitely will not) to 10 (definitely will) scale?”), and (4) *risk of liver cirrhosis* (“if you drank as much as you have tonight every week how likely is it that you will get cirrhosis of the liver in the next 15 years, on a 1 (definitely will not) to 10 (definitely will) scale?” . The key question used to test the theory was the first, with the second used to replicate the finding with alternate wording that stressed personal drunkenness as a function of that respondent’s history of alcohol use. The third question assessed the subsidiary question of how people judge the long term health risks associated with their drinking whilst intoxicated, supported by the fourth which aimed to show that the same results can apply to assessment of risk on a specific as well as a general health evaluation.

The study therefore made use of two sets of data. All those from whom BrAC was recorded were used in the ranking process. A subset of these participants also completed the risk judgement questions and it was their responses that were used to test hypotheses on the relationship between rank (relative to the larger pool of respondents) and judgements. Finally, our hypotheses were specific to drinkers in the drinking environment. Therefore respondents who yielded a BrAC of 0 μg/100 ml were not eligible for inclusion in the rank-judgement analyses, although they were included in the larger pool from which rank was determined as they were present in the same environment.

All aspects of the research presented here were scrutinised and approved by the Medical and Dental Research Ethics Committee, Cardiff University prior to data collection.

### Analytic strategy

We tested whether participants’ judgements were predicted by BrAC rank within a reference group using established statistical procedures developed for this purpose elsewhere [9, 29]. Using the whole sample of 1,862 people, we first created eight reference groups based on all combinations of gender and the four locations, on the assumption that these are the people with whom participants would compare. We included gender in the definition of reference group given the considerable differences in drinking style by gender [30]. On average, the reference group size was 231.75 respondents (min = 142, max = 343). For each person within our study group (*N* = 400), we next calculated a new variable for each individual representing the rank of an individual’s BrAC within the reference group through the formula;

1 $$ {R}_i=\frac{\left(i-1\right)}{\left(n-1\right)} $$

where the number of respondents who yielded a BrAC lower than that of the individual (*i* - 1) was compared with the total number of people within that individual’s reference group (*n* - 1) to provide a relative rank score (*R*_i_) normalised between 0 and 1. The primary test, conducted separately for each of the four outcome questions, involved simultaneously regressing outcome on both BrAC and rank BrAC, including covariates. These covariates critically included how the person’s intoxication differed from the average intoxication of those around them, in order to rule out this rival explanation of how relative comparisons are made. Only rank BrAC was hypothesised to predict each outcome.

#### Control variables

Several variables (which may have had a confounding effect) were assessed for use as covariates in a planned sensitivity analysis. Session duration might have an independent effect with those who had been drinking longest over-estimating the effects of alcohol through fatigue. Also included was the time of survey which was reduced to before and after 11 pm, a time that denotes a transition from drinking in regular pubs to nightclubs and so distinguishes between early evening and late night drinkers. Finally, FAST scores, which indicate patterns of risky drinking [27, 31], were included as these capture drinkers’ historical levels of harmful drinking.

## Results

**Fig. 1:**
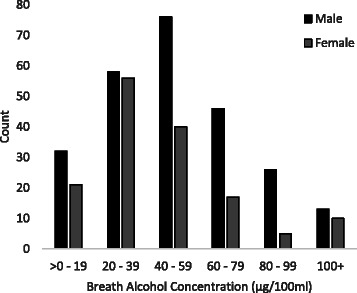
Histogram of breath alcohol scores for all respondents who yielded a BrAC greater than zero and were included in the judgement analyses (*N* = 400)

Descriptive data of participants’ responses on all measures are presented in Table 1. Figure 1 is a histogram of all respondents’ BrACs by gender, excluding those who did not yield a positive BrAC. BrAC was normally distributed for those who recorded a positive BrAC score, and ranged from less than 19 μg/100 ml breath to >100 μg. The average participant had drinking levels in excess of standard UK and US drink driving limits (35 μg/100 ml), with 10 % of the most intoxicated drinkers surveyed yielding an average BrAC of 106.4 μg/100 ml (*n* = 183). On average, participants saw themselves as moderately drunk and moderately at risk (scoring 4.5 to 6.4 on the 10 point scale). Men (mean BrAC = 52.3 μg/100 ml, *SD* = 30.0) yielded higher alcometer scores than women (mean BrAC = 43.0 μg/100 ml, *SD* = 27.8; *t* = 6.64, *p* < 0.001), and a one-way ANOVA yielded a significant main effect of location on BrAC (*F*(1, 1,860) = 44.0, *p* < 0.001). These differences by gender and location indicate that there was sufficient variation between the reference groups for rank BrAC and BrAC to provide meaningfully different measures. There was an association between rank BrAC and BrAC (*ρ* = 0.95, *p* < 0.001). We therefore used the variance inflation factor (VIF) to assess collinearity between the explanatory variables BrAC and *R*_*i*_. The VIF_i_ is given by (1 – R^2^_i_)-1 where R^2^_i_ is the R^2^ from regressing the *i*th independent variable on all other independent variables. The VIF shows how much the variance of the coefficient estimate is being inflated by multicollinearity. A VIF_i_ >10 indicates harmful collinearity [32]. In this case a VIF of 7.71 was observed.

**Table 1:** Descriptive statistics of study variables for those included in the analyses on judgement (*N* = 400)

Variable	Proportion or Mean	SD
Perceived drunkenness	4.48	1.98
Extreme drinking	4.53	2.26
Long-term health	6.40	3.21
Liver cirrhosis	6.17	3.32
BrAC (μg alcohol/100 ml breath)	47.31	27.71
Session duration (hours)	5.36	3.62
Proportion surveyed after 11 pm	0.60	-
Proportion male	0.64	-
FAST	6.18	3.49
Age (years)	26.28	8.78

Regression models were used to test hypotheses (see Table 2), with separate models for perceived drunkenness, extreme drinking, general health risks, and risk of cirrhosis of the liver. Model 1 shows that when the perceptions were regressed only on BrAC, there was a robust positive association in each. Those who were more intoxicated believed their health was at greater risk. Model 2 similarly shows that when the perceptions were regressed only on the rank of BrAC, there was again a positive association. Those who were ranked more highly in terms of intoxication believed their health was at greater risk. Model 1 explained less variance for all outcome measures than Model 2. The key test of the hypothesis is presented in Model 3, where the perceptions are jointly regressed on both BrAC and rank BrAC (controlling for the distance between the person’s BrAC and the mean of the reference group). The results are consistent across outcomes measures: only rank retains a significant positive relationship with outcomes. Taken together this suggests that when intoxicated and in a drinking environment, people’s judgements of their drunkenness (and the attendant health consequences) are only influenced by the rank of their BrAC, such that a lower relative rank corresponds with an under-appreciation of their level of intoxication. Any apparent zero-order relationship between BrAC and the judgements is apparently due to BrAC acting as a proxy for rank BrAC, as controlling for the latter removes the relationship. Model 4 presents a sensitivity analysis, repeating the tests in Model 3 whilst adjusting the exposure-outcome effect estimate for potential confounds (duration of drinking, time of assessment, gender, age, and FAST scores). The results remained the same whilst controlling for these variables.

**Table 2:** Results of the Multiple Regressions Predicting Perceptions of Drunkenness and Attendant Health Risks from BrAC, rank BrAC, and covariates

		Perceived Drunkenness			Extreme Drinking			Long-term Health			Liver Cirrhosis		
Model	Predictor	*b*	*β*	±95 % CI (b)	*b*	*β*	±95 % CI (b)	*b*	*β*	±95 % CI (b)	*b*	*β*	±95 % CI (b)
1	Intercept	2.93***		2.61, 3.25	3.29***		2.91, 3.67	5.03***		4.46, 5.60	4.84***		4.25, 5.44
	BrAC	0.03***	.45	0.03, 0.04	0.03***	.32	0.02, 0.03	0.03***	.23	0.02, 0.07	0.03***	.23	0.02, 0.04
	*R* ^*2*^ = 0.20				*R* ^*2*^ = 0.10			*R* ^*2*^ = 0.05			*R* ^*2*^ = 0.05		
2	Intercept	2.78***		2.45, 3.11	3.14***		2.75, 3.53	4.76***		4.18, 5.32	4.49***		3.88, 5.09
	***R***_***i***_	3.42***	**.47**	**2.83, 4.01**	2.77***	**.34**	**2.07, 3.47**	3.19***	**.28**	**2.14, 4.25**	3.38***	**.28**	**2.27, 4.49**
	*R* ^*2*^ = 0.22				*R* ^*2*^ = 0.12			*R* ^*2*^ = 0.08			*R* ^*2*^ = 0.08		
3	Intercept	1.68*		0.26, 3.11	1.95*		0.26, 3.65	5.59***		3.03, 8.14	5.82***		3.16, 8.48
	BrAC	−0.01	−.07	−0.03, 0.02	−0.01	−.14	−0.04, 0.01	−0.01	−.10	−0.05, 0.03	−0.02	−.13	−0.06, 0.03
	***R***_***i***_	3.90***	**.54**	**1.81, 5.00**	3.79**	**.47**	**1.31, 6.26**	4.27*	**.37**	**0.35, 8.18**	4.89*	**.41**	**0.83, 8.96**
	Group Mean	0.02	.09	−0.01, 0.05	0.03	.09	−0.01, 0.06	−0.01	-.04	−0.07, 0.04	−0.03	−.07	−0.08, 0.03
	*R* ^*2*^ = 0.23				*R* ^*2*^ = 0.12			*R* ^*2*^ = 0.08			*R* ^*2*^ = 0.09		
4	Intercept	0.87		−0.73, 2.48	0.91		−1.00, 2.82	3.71*		0.83, 6.59	3.39*		0.40, 6.37
	BrAC	−0.01	−.11	−0.03, 0.01	−0.02	−.22	−0.04, 0.01	−0.02	−.17	−0.06, 0.02	−0.03	−.21	−0.07, 0.02
	***R***_***i***_	3.78***	**.52**	**1.69, 5.87**	3.73**	**.46**	**1.25, 6.20**	4.07*	**.35**	**0.18, 7.96**	4.75*	**.40**	**0.73, 8.77**
	Group Mean	0.03	.13	−0.001, 0.07	0.03	.12	−0.01, 0.07	−0.02	−.05	−0.08, 0.04	−0.02	-.06	−0.08, 0.04
	**Duration**	0.09***	**.15**	**0.04, 0.14**	0.09**	**.14**	**0.03, 0.15**	0.09*	**.10**	**0.001, 0.18**	**0.03**	**.03**	**−0.06, 0.12**
	After 11 pm	0.30	.07	−0.05, 0.65	0.28	.06	−0.14, 0.70	0.44	.07	−0.19, 1.06	0.64	.09	−0.01, 1.29
	Male	−0.24	−.06	−0.66, 0.18	−0.20	−.04	−0.70, 0.30	0.04	.01	−0.70, 0.78	−0.20	−.03	−0.97, 0.57
	FAST	−0.01	−.02	−0.06, 0.04	0.07*	.11	0.01, 0.14	0.16**	.17	0.06, 0.25	0.21***	.22	0.12, 0.31
	Age (years)	0.003	.01	−0.02, 0.02	−0.001	.00	−0.02, 0.02	0.03	.08	−0.01, 0.06	0.04*	.10	0.001, 0.07
	*R* ^*2*^ = 0.26				*R* ^*2*^ = 0.16			*R* ^*2*^ = 0.12			*R* ^*2*^ = 0.14		

Note: * *p* < .05; ** *p* < .01; *** *p* < .001

To test whether people were more influenced by those who ranked above or below them, the regressions in Model 2 were re-run but with the rank amongst the eight location by gender categories formed through a version of Eq. 1 with additional parameters:

2 $$ S{R}_i=0.5+\frac{\left(i-1\right)-\eta \left(n-i\right)}{2\left[\left(i-1\right)+\eta \left(n-i\right)\right]} $$

through Eq. 2 it is possible to find the value of *η* that explains any variance in the outcome variables (perceived risk and intoxication) best; *η* > 1 indicates an upward bias and that drinkers are more influenced by more intoxicated drinkers whereas *η* < 1 indicates a downward bias and that respondents are more influenced by more sober drinkers [33]. To determine the value of *η* an iterative process was used in which values of *η* from 0.01 to 5 in 0.01 steps were regressed onto outcomes. The value of *η* yielding the greatest R^2^ value was then selected for each outcome. For all judgements respondents were more influenced by those who were more sober: perceived drunkenness *η* = 0.70, extreme drinking *η* = 0.14, long-term health *η* = 0.17 and liver cirrhosis *η* = 0.21. In other words introducing sober people into a drinking environment would be predicted to have greater impact on judgements, making people feel more at risk and more intoxicated, compared to the effect on decreasing feelings of riskiness one might expect if very intoxicated people were introduced into the environment. It appears that drinkers are more self-aware of their own level of intoxication when in the presence of those who are sober.

## Discussion

The study sheds new light on how people judge their drunkenness and the health consequences of their drinking whilst actually intoxicated in real world drinking environments. In such situations the relationship between actual intoxication and drinking perceptions was accounted for by how the person’s consumptions ranked amongst others. Thus, using the same cognitive processes as used in other psychophysical judgements, perceptions of one’s intoxication appear to arise from comparison to others. People are also more influenced by those who are more sober than them, relative to those who are more intoxicated.

The research builds on previous social norms work by indicating that people’s judgements of their alcohol use depend on their perception of how intoxicated other people are. The study expands on knowledge by: (a) extending the social norms perspective to judgements made whilst intoxicated in drinking environments; (b) showing that in such settings people are influenced by the actual rather than imagined behaviour of others; (c) showing that in this context people are more influenced by those who drink less (whilst in social norms research using sober subjects, people tend to underestimate their relative drinking, suggesting a greater focus on those who drink more); and (c) consistent with one other paper [9], suggesting that when comparing to others, people are influenced by the rank position relative to others.

The finding that people are specifically sensitive to rank position suggests that a basic evolutionary mechanism may be implicated. Animals, ranging from crayfish to monkeys, are generally very sensitive to rank position within a hierarchy [34], and in the clinical literature there is increasing realisation that mental disorders may partially arise from a mis-regulation of mechanisms that were adaptive in the evolutionary past [35]. Specifically, whilst high sensitivity to rank position (including hypervigilance, lower appetite, reduced sexual behaviour, and general withdrawal amongst those of low rank) would have conferred a survival advantage in the evolutionary past, such hard wired tendencies may influence people in negative ways in modern society [36–39]. On the basis of the results described here, we suggest that an inbuilt sensitivity to rank position amongst others can maladaptively lead people to assume they are less drunk and at risk than they actually are if they rank low amongst other drinkers. Such rank sensitivity may also explain why drinking increases in a society; if everyone drank another 10 units per week, no one would believe themselves to be at more at risk of alcohol related disorder as their rank positions would remain the same. Future research could usefully examine whether this rank sensitivity can be harnessed through intervention; asking whether, for example, telling people how they rank amongst a broader reference population (e.g., “you are in the top 5 % of drinkers”) would decrease drinking more than the dominant social norms intervention approach of telling people how much more they drink than the average or typical person. Consistently, the “nudge” [40] approach of behavioural economics suggests that greater behavioural change occurs when information is presented in line with people’s natural ways of processing information. Given that people are influenced by more sober others, a further “nudge” maybe achievable by increasing the mix of consumers in drinking environments, incentivising the presence of more sober people, for example through soft drink pricing, or by attracting a more diverse population of entertainment seekers, or by introducing a range of ‘capable guardians’ such as street pastors, taxi marshalls and city ambassadors who, in addition to their formal roles, would also act as sober comparators with which at risk drinkers would compare themselves. More research is needed here since research on behavioural strategies to reducing misuse at the point of sale is very limited [25]. However, it is clear from the findings of the present study that alcohol harm reduction strategy should capitalise on knowledge that people in drinking environments make decisions to drink more on the basis of their observation of people around them.

All studies that study alcohol use in context face numerous difficulties, challenges and are therefore limited. This is usually offset by the considerable advantages of studying behaviour in situ. Never-the-less, this paper has several limitations. First, the operationalization of a social network here assumes those in the same environment who are consuming alcohol influence one another. This may not be consistent with the generic social network approach, in as far that many of those in this study probably have no social relationship. However, those surveyed will have been aware of other people’s presence at least visually. Given the marked effect of alcohol on appearance, such as alcohol-related ataxia, visual cues will play an important role in the formation of judgements. This does warrant further investigation and studies might consider the ecology of influences from immediate social groups through to those in the same venue and environment. Furthermore, the measure for rank intoxication could potentially be subject to less measurement error than the more objective BrAC and this is a concern when measures are correlated [41], as is the case here. However, theoretically the expectation that rank outperforms absolute measures is robust [11, 12, 15, 16] and in areas where measurement area is less likely a factor (such as large scale comparisons between rank income and absolute income in predicting well-being [29]) rank performs similarly. We are therefore confident that the effects reported here are genuine. Nevertheless, the matter warrants further attention and research could partially address this through manipulating the context in which measures are taken such that the expectation is that rank would systematically vary.

## Conclusion

While this paper is primarily interested in the manner in which context, or the reference set comprised of other drinkers, informs judgements of being at risk from alcohol consumption, there are obvious links with the traditional literature on perceptions of intoxication generally. For example, self-rated intoxication becomes less accurate as the level of intoxication increases [42]. Certainly the factors that govern drinkers’ choices regarding further alcohol consumption are many and intertwine in complex ways; possibly only a few are amenable to intervention. The environment is one factor that is modifiable and it is encouraging to note that changes in licensed premises density correspond with changes in the prevalence of outcomes, such as violence and excessive alcohol consumption, that are related to both alcohol and poor decision making [43]. A high density of licensed premises would mean fewer alternative venues that do not sell alcohol and therefore a greater density of drinkers in those environments. A logical next step is therefore to consider the distribution of drinkers in environments varying by premises density as a potential feature that links alcohol use with excessive consumption and harm.

## Abbreviations

BrAC, breath alcohol content; CI, confidence interval; FAST, fast alcohol screening test; ml, millilitre; Ri, relative rank score; VIF, variance inflation factor; μg, micrograms
